# Marker discovery in the large

**DOI:** 10.1093/bioadv/vbae113

**Published:** 2024-07-27

**Authors:** Beatriz Vieira Mourato, Ivan Tsers, Svenja Denker, Fabian Klötzl, Bernhard Haubold

**Affiliations:** Research Group Bioinformatics, Max-Planck-Institute for Evolutionary Biology, 24306 Plön, Schleswig-Holstein, Germany; Research Group Bioinformatics, Max-Planck-Institute for Evolutionary Biology, 24306 Plön, Schleswig-Holstein, Germany; Research Group Bioinformatics, Max-Planck-Institute for Evolutionary Biology, 24306 Plön, Schleswig-Holstein, Germany; Universität zu Lübeck, Lübeck, Schleswig-Holstein, Germany; Cambridge CB2 1LB, United Kingdom; Research Group Bioinformatics, Max-Planck-Institute for Evolutionary Biology, 24306 Plön, Schleswig-Holstein, Germany

## Abstract

**Motivation:**

Markers for diagnostic polymerase chain reactions are routinely constructed by taking regions common to the genomes of a target organism and subtracting the regions found in the targets’ closest relatives, their neighbors. This approach is implemented in the published package Fur, which originally required memory proportional to the number of nucleotides in the neighborhood. This does not scale well.

**Results:**

Here, we describe a new version of Fur that only requires memory proportional to the longest neighbor. In spite of its greater memory efficiency, the new Fur remains fast and is accurate. We demonstrate this by applying it to simulated sequences and comparing it to an efficient alternative. Then we use the new Fur to extract markers from 120 reference bacteria. To make this feasible, we also introduce software for automatically finding target and neighbor genomes and for assessing markers. We pick the best primers from the 10 most sequenced reference bacteria and show their excellent *in silico* sensitivity and specificity.

**Availability and implementation:**

Fur is available from github.com/evolbioinf/fur, in the Docker image hub.docker.com/r/beatrizvm/mapro, and in the Code Ocean capsule 10.24433/CO.7955947.v1.

## 1 Introduction

Genetic markers are regions that occur in the genomes of all targets and, ideally, nowhere else. In modern workflows, “nowhere else” is achieved by comparing marker candidates to a sample of genomes taken from phylogenetic neighbors (SantaLucia *et al.* 2020). Karim *et al.* (2019) implemented this principle in their program Uniqprimer and used it to design diagnostic primers for the bacterial pathogen that causes soft rot in potato, *Dickeya dianthicola*. Uniqprimer is based on the fast genome aligner nucmer (Marçais *et al.* 2018) to intersect the target genomes and subtract the neighbors.

Two years after the publication of Uniqprimer, Beran *et al.* (2021) published a tool based on fixed-length exact matches, or k-mers, for finding diagnostic regions. Their program, KEC for k-mer elimination by cross-reference, picks target regions that contain no k-mer matches to neighbors. Looking up k-mers amounts algorithmically to hashing, which makes KEC highly efficient. Its authors went on to design markers for *Xanthomonas euvesicatoria*, the bacterium responsible for bacterial spot disease in tomatoes (Beran *et al.* 2023). In addition, Wang *et al.* (2024) have used KEC to design markers for *Exserohilum turcicum*, a fungus that causes northern corn leaf blight.

Haubold *et al.* (2021) used an alternative approach to marker discovery based on maximal matches, which they looked up using enhanced suffix arrays. For random sequences, the length distribution of maximal matches is known (Haubold *et al.* 2009), which allows the efficient identification of regions with effectively random matches, the desired marker regions without homology elsewhere. Haubold *et al.* (2021) implemented this approach in the program Fur for finding unique regions.

Fur extracts markers in three steps by (i) subtracting the neighbors from a target representative, *r*, (ii) intersecting the remainder of *r* with the other targets, and (iii) checking the intersection for residual neighbor material with Blast (Haubold *et al.* 2021).

The first step is based on all matches between *r* and the neighbor sequences. Up to version 3.2 of Fur, these matches were obtained in the single-step algorithm developed by Pirogov *et al.* (2019) and implemented in their program macle, which calculates a suffix array from *r* and all neighbors.

This matching procedure is fast and easy to implement, but it has two disadvantages. The first is that matches within the target cannot be distinguished from matches elsewhere. In other words, a repeat in the target would not be included among the markers even if it was absent from the neighbors.

The second and often more serious disadvantage is that the memory requirement of macle, and hence also of Fur, scales with the number of nucleotides in the neighborhood, which can easily contain hundreds of bacterial genomes. At roughly 65 bytes of RAM per bp, this means that the analysis of targets with large neighborhoods may require specialized hardware, or fail altogether.

Both of these disadvantages are mitigated by our new version of Fur, where the call to macle is replaced by a variant of the calculation of the matching statistics for two strings. The concept of matching statistics was first defined by Chang and Lawler (1994) as the lengths of the longest substrings of a query, *q*, that match somewhere in a reference, *r*. Put more formally, for 1≤i≤|q|, the matching statistics ${\text{ms}}_{q,r}[i]$ is the length of the longest prefix of q[i…] that occurs somewhere in *r*. The original algorithm for calculating the matching statistics of *q* with respect to *r* was based on a suffix tree of *r*. Abouelhoda *et al.* (2004) replaced the suffix tree by the enhanced suffix array, and interest in methods for fast computation of matching statistics has remained strong, see e.g. the recent paper by Lipták *et al.* (2024) and references therein.

Instead of calculating the full matching statistics, we take the simplifying shortcut of determining match lengths only at the points where new matches start and interpolate the remaining match lengths. For this purpose, our new algorithm iterates over the neighbors and indexes each one in turn. Each index is a suffix array enhanced to function as a suffix tree. One target, e.g. the shortest, is matched into that tree starting at the root and the first target position. At the first mismatch, the length of the match is noted and the matching repeats from the first position beyond the mismatch. Whenever a longer match is found than the one noted so far, the longer one is kept.

With this algorithm, matches can only occur between targets and neighbors, not within a target. And since the memory requirement of constructing an enhanced suffix array is linear in the input, the new algorithm requires memory proportional to the length of the longest neighbor. In other words, for a given organism, the memory requirement becomes independent of the number of neighbor sequences, allowing the program to run on a laptop where it previously might have run out of memory on a server. This opens the way for picking markers from samples of bacterial genomes of any size, marker discovery in the large.

While an efficient and accurate selection program like Fur is necessary for large-scale marker discovery, in practice, we found that it is not sufficient. This is due to the difficulty of picking suitable genomes for marker selection and of assessing the markers returned. For input, we developed the package Neighbors to find provisional target and neighbor genomes from the NCBI taxonomy (Schoch *et al.* 2020), which we then sort into monophyletic targets and their neighbors.

We assess the quality of markers by picking the best pair of PCR primers from them. Then we calculate the specificity and sensitivity of these primers by running *in silico* PCR against the non-redundant collection of nucleotide sequences provided by the NCBI, nt. These two steps, primer design and primer assessment, are implemented in our package Prim.

To summarize, our workflow for marker discovery in the large consists of three steps, which are supported by three software packages:

1. find target and neighbor genomes with Neighbors;
2. find markers with Fur; and
3. find and evaluate primers with Prim.

In the following, we first explain these packages in more detail. Then we apply Fur and its alternative KEC to simulated data to compare their resource consumption and accuracy. Next, we apply Fur to a list of 120 reference prokaryotes supplied by the NCBI. According to its authors, this list is a “small curated subset of really good and scientifically important prokaryotic genomes.” For each taxon, we attempt to extract markers and to design primers. We report in detail on the 10 taxa with the largest number of sequenced genomes.

## 2 Methods

Our tools for marker discovery are written in Go and run under the Unix command line. We explain this software before describing how we simulated test data and analyzed 120 reference prokaryotes. Throughout this description we mention six software packages, Fur, Neighbors, Prim, Stan, Biobox, and Mapro, which are available from our github page,

github.com/evolbioinf/*ext*

where *ext* is fur, neighbors, prim, stan, biobox, or mapro.

### 2.1 Marker discovery

#### 2.1.1 Fur

The package Fur is centered on two programs, makeFurDb for generating a database of target and neighbor sequences, and fur for extracting the markers from the database. We describe version 4.2, where we iterate over the neighbors, *n_i_*, and convert each one into an enhanced suffix array, esa, in turn (Algorithm 1, line 2). The suffix array underlying the esa is calculated using the divsufsort algorithm (Fischer and Kurpicz 2017) as implemented in libdivsufsort (Mori 2015). To obtain the final esa, the suffix array is enhanced by its longest common prefix array, lcp, and its child array (Ohlebusch 2013, Section 4.3.4). These additions turn the esa into an lcp-interval tree, an efficient version of a suffix tree (Ohlebusch 2013, Section 4.3).

Then we traverse the target representative, *r*, in steps of matching prefixes. Prefix matching is implemented in the method MatchPrefix of the enhanced suffix array, called in line 5 of Algorithm 1. This is based on a traversal of the lcp-interval tree of *n_i_* encapsulated in our esa as described by Ohlebusch (2013, p. 119) in his Algorithm 5.2. MatchPrefix returns the length of the match, ℓ. If the entry in the array of match lengths, ml, at the start of the match, *j*, is less than the new match length, we update it to the greater length (Algorithm 1, line 6).

Algorithm 1. Iterative computation of match lengths.

**Require:** *r* ▹ target representative

**Require:**$N=\{n_{1},n_{2},\ldots ,n_{m}\}$ ▹ set of *m* neighbor sequences

**Ensure:** ml ▹ array of match lengths along *r*

1: **for**i←1,m**do**

2:   $\text{esa}\leftarrow \text{getEsa}(n_{i})$

3:    j←1

4:    **while**j≤|r|**do**

5:      ℓ←esa.MatchPrefix(r[j…])

6:      ml[j]←max(ml[j],ℓ)

7:      j←j+ℓ+1

8:    **end while**

9: **end for**

Algorithm 1 lends itself to parallelization. In our implementation in the program makeFurDb we read all neighbors, divide them by the number of threads available, and process the sets of neighbors in parallel. By default, the number of threads is equal to the number of CPUs, though the user can set that number to some other value. For example, the user might wish to minimize memory consumption by setting the number of threads to 1.

The array of match lengths returned by Algorithm 1, ml, is completed in a left-to-right traversal by setting ml[i+1]←ml[i]−1 wherever ml[i+1]<ml[i]−1. Then, ml is subjected to a sliding window analysis in fur to extract all regions with matches that are indistinguishable from random based on the null distribution of match lengths derived by Haubold *et al.* (2009). These regions are the desired target segments without homology among the neighbors. We intersect them with all targets and check the remainder with Blast against the neighbors to get the putative markers (Haubold *et al.* 2021).

#### 2.1.2 Neighbors

**Figure 1.**
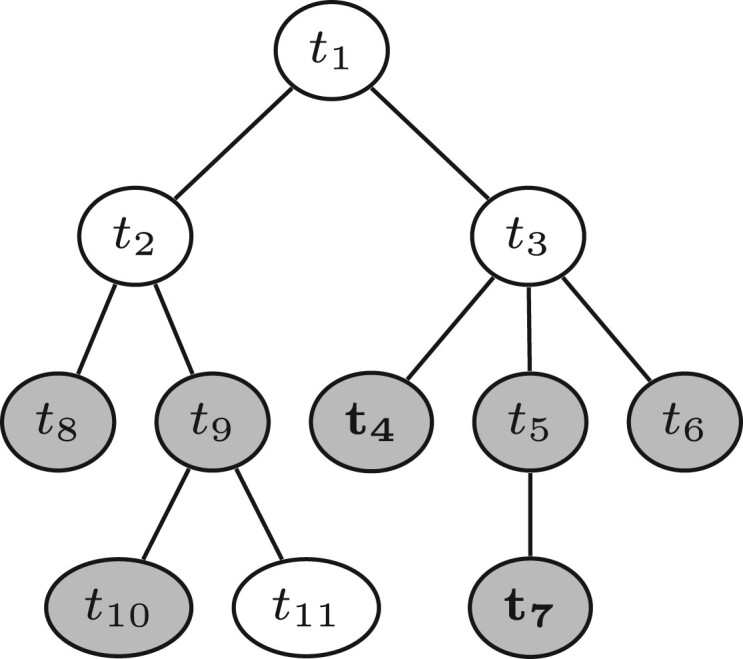
Toy taxonomy, where taxa with sequenced genomes are shown in gray.

The target and neighbor genomes required for marker discovery were found using the Neighbors package. It initially picks the genomes from a taxonomy linked to genome accessions. Consider the toy taxonomy in Fig. 1. Each taxon has a taxon-ID, *t_i_*; it may also have one or more accessions of genome assemblies associated with it, in this case it is marked gray. Let us now consider target taxa *t*_4_ and *t*_7_ shown in bold in Fig. 1. By following their lineages toward the root, we find that their most recent common ancestor is *t*_3_. This implies that the complete target list consists of the known targets *t*_4_ and *t*_7_, plus the new targets *t*_3_, *t*_5_, and *t*_6_. Notice that we included internal and terminal nodes in our target list, as both types of nodes may be associated with genome sequences. So, we can write our taxonomic target set as:

T=s(m),

where *m* is the most recent common ancestor of the targets and *s*(*m*) the set of nodes in the subtree rooted on it. Among the targets in our example, *t*_4_, *t*_5_, *t*_6_, and *t*_7_ are associated with one or more genome assemblies.

Starting from *m*, we find the closest taxonomic neighbors by climbing to its parent, *p*(*m*). All children in the subtree rooted on *p*(*m*) minus the targets and *p*(*m*) itself make up the set of provisional neighbors, which we can write as:

N=s(p(m))∖T∖p(m).

As we already observed, the most recent common ancestor in our example in Fig. 1 is *m* = *t*_3_. Its parent is *t*_1_. This gives us the neighbor taxa *t*_2_, *t*_8_, *t*_9_, *t*_10_, and *t*_11_. Among these, we consider the assemblies associated with *t*_8_, *t*_9_, and *t*_10_.

To get from taxonomic targets and neighbors to monophyletic targets and their neighbors, we calculate the phylogeny of the genomes just found in the taxonomy. In that phylogeny the desired target clade maximizes the number of taxonomic targets inside and the number of neighbors outside. It is easy to visually pick this clade from a small phylogeny. However, in real marker searches we may be dealing with hundreds of genomes, which makes visual inspection of their phylogeny difficult. Instead, our Neighbors package provides the program fintac, which reads a phylogeny and returns the clade that maximizes the sum of the number of targets inside and the number of neighbors outside.

Neighbors is based on the taxonomy obtained from a dump of the NCBI taxonomy (Schoch *et al.* 2020) and genome lists also provided by the NCBI. The database dump is available from

*ncbi*/pub/taxonomy/taxdump.tar.gz

where *ncbi* is ftp.ncbi.nlm.nih.gov. The genome lists are posted as three separate files for prokaryotes, eukaryotes, and viruses,

*ncbi*/*genomes*/*taxon*.txt

where *genomes* is genomes/GENOME_REPORTS and *taxon* is prokaryotes, eukaryotes, or viruses. The database dump and the genome lists are frequently updated. In our analysis, we used the version we downloaded on 21 December 2023.

The taxonomy files are converted to a relational database, which is built in less than 20 s using the program makeNeiDb. The database is managed with sqlite3, so it can be directly queried. However, the most important queries are canned in programs, notably neighbors for picking taxonomic target and neighbor genomes. Querying is quick because we index the tables of the underlying database; a typical run of neighbors takes less than 5 ms.

#### 2.1.3 Prim

The package Prim contains programs for generating and checking primers. Primers are generated by calling primer3 (Untergasser *et al.* 2012). They are checked by *in silico* PCR on a Blast database with taxonomic data. These taxonomy-based primer scores are then checked again using evolutionary distances.

### 2.2 Simulated data

We simulated sets of target and neighbor sequences using our package Stan. It simulates a coalescent with the deepest split between the target and the neighbor clades. Then it generates sequences with mutations along this genealogy and adds “markers” by deleting regions from all neighbors. These sequences are written in FASTA format to directories ready for analysis with a program like Fur or KEC.

#### 2.2.1 Accuracy

We quantified the accuracy of Fur using a measure developed in gene prediction (Haubold and Wiehe 2006, p. 120f). Let $t_{p}$ be the number of true-positive nucleotides, $t_{n}$ the number of true-negatives, $f_{p}$ the number of false-positives, and $f_{n}$ the number of false negatives, then we write the accuracy as:

(1) $$A=\frac{t_{p}t_{n}-f_{p}f_{n}}{\sqrt{\left(t_{p}+f_{p})(t_{n}+f_{n})(t_{n}+f_{p})(t_{p}+f_{n}\right)}}.$$

#### 2.2.2 Resource consumption

We measured single-threaded resource consumption with the command /usr/bin/time on a laptop running the Windows Subsystem for Linux under Windows 11 equipped with 32 GB of RAM and 16 logical 12th gen Intel i7 processors clocked at 2.5 GHz.

### 2.3 Diagnostic primers

The list of 120 reference prokaryotes we used is available from

*ncbi*/*genomes*/prok_reference_genomes.txt.

We took the type strain of each taxon, looked up its parent taxon, and used that as target. We downloaded the genomes returned by neighbors for that target with the datasets tool provided by the NCBI, where we excluded “atypical” genomes and genomes with incomplete assemblies. From these genomes we calculated pairwise distances using phylonium (Klötzl and Haubold 2020), which we summarized into a midpoint-rooted phylogeny using the programs nj and midRoot from the Biobox package. We labeled the internal nodes of this phylogeny with land (Neighbors) to prepare it for finding the target clade with fintac (Neighbors). The target clade implies the final set of targets and neighbors, from which we picked the markers with Fur.

From the markers we extracted the highest scoring pair of primers for an amplicon between 70 and 150 bp, and calculated sensitivity and specificity of the primer pair by *in silico* PCR using scop (Prim). The *in silico* PCR was based on Blast searches against the non-redundant collection of nucleotide sequences, nt, and its corresponding taxonomy, which we downloaded on 24 November 2023 from

*ncbi*/blast/db.

This taxonomy may not reflect the true phylogeny, so we correct the provisional primer scores of scop using evolutionary distances estimated from DNA sequences, as implemented in cops (Prim). As threshold for correcting the primer scores with cops, we used twice the cumulative branch length from the type strain to the parent of the target clade, which we looked up with climt (Neighbors). The complete work flow for analyzing the 120 prokaryotes is presented as a documented script in the Mapro package, which can be run in the Docker container beatrizvm/mapro.

## 3 Results

### 3.1 Resource consumption

We investigated the time and memory consumption of the two central programs of the Fur package, makeFurDb for constructing the database from which the markers are then picked with fur. We compared our new memory-efficient version that implements Algorithm 1 with its predecessor (Haubold *et al.* 2021) and with KEC (Beran *et al.* 2021), and varied both sequence length and the number of neighbor sequences. KEC version 1 was run on both strands of the DNA sequence and with default k-mer length 12, which is its most efficient setting according to the section on resource consumption of the program’s github page (Beran 2021). All measurements were done in single-threaded mode.

**Figure 2.**
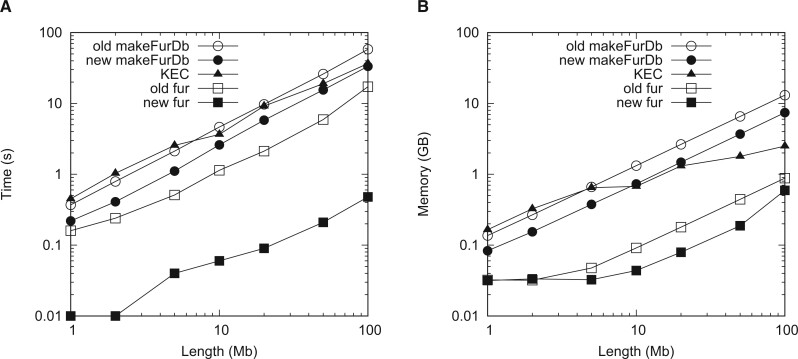
Time (A) and memory (B) consumption of the old and new makeFurDb, fur, and KEC as a function of sequence length.

When investigating sequence length, we simulated one target and one neighbor of lengths varying from 1 to 100 Mb. As shown in Fig. 2A, the run time of both versions of makeFurDb and fur and of KEC scales approximately linearly with sequence length. The new makeFurDb takes roughly 0.3 s per Mb, which is twice as fast as the old version with 0.6 s per Mb, and slightly faster than KEC. The new fur takes roughly 0.6 ms per Mb, over 25 times faster than the old version with 17.5 ms per Mb.

The memory requirements of both versions of makeFurDb and fur shown in Fig. 2B also scale approximately linearly with sequence length. The new makeFurDb requires 74 bytes per bp, just over half of the 131 bytes per bp required by the old. The memory requirement of 131 bytes per bp seems to be at odds with the requirement of 65 bytes per bp stated in Section 1. However, the old makeFurDb indexed the target *and* the neighbor, hence the apparent doubling of the memory requirement. The memory requirement of KEC is identical to that of the old makeFurDb up to 5-Mb-long sequences, whence the memory requirement of KEC tails off. The new fur requires 6 bytes per bp, a third less than the 9 bytes per bp for the old version. However, this difference appears to diminish with sequence length.

**Figure 3.**
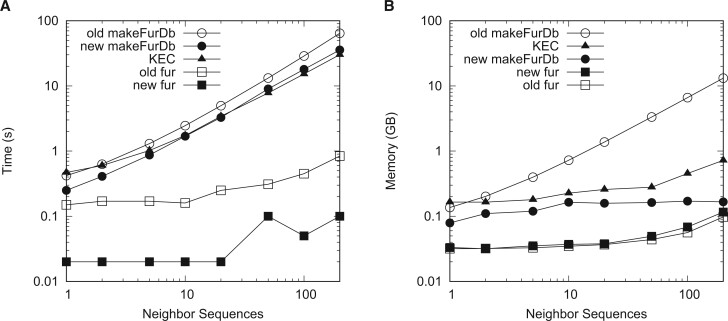
Time (A) and memory (B) consumption of the old and new makeFurDb, fur, and KEC as a function of the number of 1-Mb neighbor sequences.

To investigate the effect of the number of neighbor sequences on resource consumption, we simulated one target and between 1 and 200 neighbors, each 1 Mb long. Figure 3A shows that the run time is linear in the number of neighbors for samples of at least 20 neighbor sequences. The new makeFurDb takes 35.7 s for 200 sequences, roughly half the run time of the old makeFurDb, and 30% more than KEC.

As to memory consumption, Fig. 3B shows that makeFurDb uses 80 times less memory for the 200 neighbor sequences than the old version and a quarter of the memory used by KEC. Also notice that the memory consumption of the new makeFurDb is constant in the number of neighbors, and that of KEC is almost constant. In other words, as the neighborhood grows, so does the difference between the memory requirement of the old makeFurDb on the one hand and the new makeFurDb and KEC on the other.

### 3.2 Accuracy

**Figure 4.**
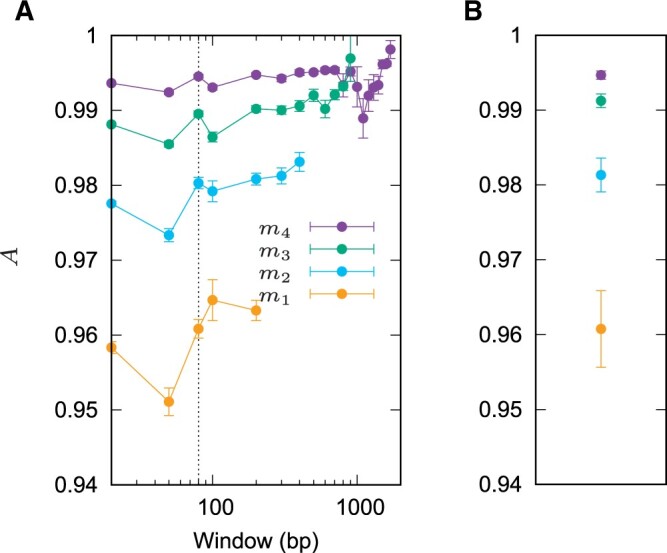
The accuracy, *A*, as defined in equation (1), of Fur (A) and KEC (B) for four simulated markers, $m_{1},\ldots ,m_{4}$ with lengths 200,400,800,1600 bp, respectively in samples of 10 target and neighbor “genomes” each 5 Mb long. Plot points represent mean±SEM of 100 simulations. The accuracy of Fur is shown as a function of window length, the dotted line is the default length. There is no sliding window used in KEC and hence its accuracy is shown as constants with the same color code for marker length drawn on the same *y*-scale.

Having established the efficiency of Fur and KEC, we quantified their accuracy by simulating sets of 10 targets and 10 neighbors each 5 Mb long. As markers we generated one centered deletion in the neighbors of lengths 200, 400, 800, and 1600 bp. Then we used Fur to detect these markers with sliding windows of lengths ranging from 20 to 1700 bp. Our measure of accuracy, *A*, defined in equaiton (1), can be thought of as a combined measure of sensitivity and specificity. Figure 4A shows that the accuracy of Fur is greater than 0.95 throughout, with short error bars. Moreover, longer markers tend to be detected with greater accuracy than shorter markers. The graphs for Fur in Fig. 4A break off at the point at which no marker material is detected any more in 100 iterations. For example, the 200 bp marker cannot be detected with windows longer than 200 bp, while the 1600 bp marker cannot be detected with windows longer than 1700 bp. The default window length, 80 bp, is chosen to harmonize with the minimum requirement of 100 nucleotides in a unique window.

In contrast to Fur, KEC uses no sliding window, so we plotted its accuracy in Fig. 4B along the same *y*-axis. The accuracy of KEC is similar to that of Fur in this simulation, though for longer markers the results of KEC vary less.

### 3.3 Marker discovery

Out of the 120 reference prokaryotes analyzed, we found markers for 96 taxa. These marker sets ranged from 158 bp to 3.8 Mb and are contained in our Code Ocean capsule. From these 96 markers sets, we extracted primers for 89. Table 1 lists the 10 most sequenced targets among the primed taxa ordered by the number of target genomes. This ranges from 675 targets for *Pseudomonas aeruginosa* to 141 for *Burkholderia pseudomallei*. The number of neighbor genomes ranges from 1460 for *Staphylococcus epidermidis* to just 1 for *Mycobacterium tuberculosis*.

**Table 1.** The number of target genomes, $n_{t}$, and neighbor genomes, $n_{n}$, for the 10 most sequenced reference prokaryotes.

#	Species	Type strain	$n_{t}$	$n_{n}$
1	*Pseudomonas aeruginosa*	PAO1	675	23
2	*Bordetella pertussis*	Tohama I	659	225
3	*Mycobacterium tuberculosis*	H37Rv	437	1
4	*Enterococcus faecium*	DO	332	317
5	*Lactiplantibacillus plantarum*	WCFS1	247	15
6	*Enterococcus faecalis*	V583	228	421
7	*Streptococcus pneumoniae*	R6	178	1074
8	*Staphylococcus epidermidis*	ATCC 12228	154	1460
9	*Salmonella enterica*	CT18	151	1050
10	*Burkholderia pseudomallei*	K96243	141	61

From the 10 most sequenced targets we extracted between 1.0 and 92.6 kb of marker material (Table 2). The markers were sprinkled with between 1 and 10,157 Ns, positions that are polymorphic in at least one genome contained in the pileup of targets. Fur marks polymorphic positions as Ns to allow software for primer design to later avoid them.

**Table 2.** Markers for the 10 most sequenced reference prokaryotes and the number of polymorphic sites (Ns) they contain.

#	Species	bp	Ns
1	*Pseudomonas aeruginosa*	65 063	10 157
2	*Bordetella pertussis*	1033	1
3	*Mycobacterium tuberculosis*	13 967	272
4	*Enterococcus faecium*	29 380	3759
5	*Lactiplantibacillus plantarum*	30 625	4241
6	*Enterococcus faecalis*	92 560	7624
7	*Streptococcus pneumoniae*	5899	259
8	*Staphylococcus epidermidis*	34 528	4322
9	*Salmonella enterica*	1246	144
10	*Burkholderia pseudomallei*	29 242	1963

From the markers we extracted the primers shown in Table 3. Their penalty ranges from 0.03 for *Streptococcus pneumoniae* to 2.84 for *Lactiplantibacillus plantarum*. The primer penalties hint at their eventual performance *in vitro*. However, more relevant at this stage is the *in silico* sensitivity and specificity of each primer pair. As shown in Table 3, the sensitivity varied only between 0.99 and 1.0. Similarly, the specificity was perfect throughout with one exception, *Salmonella enterica* typified by strain CT18, which had a specificity of only 0.72.

**Table 3.** Forward (f) and reverse (r) primers for the 10 most sequenced referenced bacteria, including their penalty (Pen.), sensitivity (Sen.), and specificity (Spe.).

#	Species	Forward	Reverse	Pen.	Sen.	Spe.
1	*Pseudomonas aeruginosa*	TGTATGGATGCTAGGTACCC	CACGGATGAACCAATACGAA	0.34	1.00	1.00
2	*Bordetella pertussis*	GAAAGCGGTTGATCAATCCT	CGGTTCCATCATCTGCTAAG	0.13	0.99	1.00
3	*Mycobacterium tuberculosis*	GCCTGTTGTAAAGGTAACGT	GAAGTTCAACTGCTGGTGAT	0.05	1.00	1.00
4	*Enterococcus faecium*	CCCGCAAATACTAGAGGTTC	TGCAGTCAGCTATGCAATAC	0.44	1.00	1.00
5	*Lactiplantibacillus plantarum*	CTGTACTTGCATGCGAACTA	TGCTAGTTTCATGCTTGTCG	0.57	1.00	1.00
6	*Enterococcus faecalis*	ATTTAGGAGAGTGAGGCGAA	GAGAGACCGAAAAGCCAATT	0.08	1.00	1.00
7	*Streptococcus pneumoniae*	AAGTTGAAGAAGCGGAAGAC	ATGCATGCAGAAGACCAAAT	0.03	1.00	1.00
8	*Staphylococcus epidermidis*	TGATGTTTGATGCATGTACAGT	GAATGAAGCGACTCGTTGAA	2.84	1.00	1.00
9	*Salmonella enterica*	TGTTGTAGTTGAAGGCCATG	ATTTGAGCATAGCGGTGATC	0.06	1.00	0.72
10	*Burkholderia pseudomallei*	TGAGCCGTTTAGATCTCCTT	GCGTATATCGTATGCAGGAC	0.06	1.00	1.00

To further investigate our primers, we looked up the coordinates of their amplicons in the genomes of their respective type strains. These coordinates are listed in Supplementary Table S1 together with the annotations that intersect them. Six out of the 10 amplicons are part of genes that encode a “hypothetical protein.” However, in *S.enterica* that hypothetical protein is part of a lysogenic bacteriophage, a mobile genetic element. This might explain the specificity of only 0.72 of the primer pair for *S.enterica* (Table 3). For that species, we found 67 genomes in nt that contained the marker but were rather distantly related to the type strain with distances between 14% and 15%, 10 times more than the target threshold of 1.4%. This observation of a marker in the “wrong” genetic background is consistent with horizontal gene transfer via the bacteriophage.

## 4 Discussion

Limberis and Metcalfe (2023) recently published a program for designing multiplex PCR primer sets. They demonstrated their program by designing and testing eight primer pairs to target eight drug-resistant genes in *M.tuberculosis*. This taxon is also included in the 10 we have concentrated on in this study (Table 1). The approach of Limberis and Metcalfe (2023) to designing diagnostic primers is an example of the traditional candidate gene approach. This method involves starting with the biology of an organism, selecting markers—such as genes encoding toxins or antibiotic resistance—and designing primers based on these markers. These primers are then tested by *in silico* PCR and *in vitro*. The main limitation of the candidate gene approach, as Limberis and Metcalfe (2023) themselves point out, is that the user needs to supply the markers. Haubold *et al.* (2021) developed Fur for quickly finding markers from whole-genome sequences without referring to genes or any other annotation.

Like other modern programs for marker discovery (Karim *et al.* 2019, Beran *et al.* 2021), Fur takes as input a sample of monophyletic target genomes and a sample of closely related neighbor genomes and returns the regions common to the targets that are absent from the neighbors. In principle, this allows the extraction of markers from any target organism for which a suitable collection of target and neighbor genomes is available. After almost three decades of whole-genome sequencing in bacteria, many pathogens fall into that category. However, two obstacles have in the past hampered marker discovery in the large with Fur. The first was specific to Fur, its excessive memory requirement. The second was the general lack of supporting software, making it difficult to find target and neighbor genomes and to evaluate markers.

Algorithm 1 solves Fur’s memory problem by dividing the indexing step and storing the intermediate results. This is feasible because when searching for the maximum length among a set of matches, the order in which the matches arrive is irrelevant, i.e. max(max(a,b),c)=max(a,max(b,c)). Dividing the index computation reduced the memory requirement of Fur from proportional to the size of the neighborhood to proportional to the longest neighbor, which even makes it competitive with a program based on k-mers like KEC (Fig. 3B). And because the run time of constructing suffix arrays is almost linear in the length of the input string, we expected our new algorithm to be as fast as its predecessor. In fact, it is faster (Figs 2A and 3A), which we attribute to the replacement of the call to the external program macle by internal function calls. When comparing the speed of the new Fur to KEC, makeFurDb is slower for samples of long or many sequences, but fur is much faster (Figs 2A and 3A). This emphasizes the importance of separating index construction from marker extraction.

While the current efficiency of Fur is commensurate with our goal of large-scale marker discovery, there remains room for improvement. In particular, our iterative calculation of match lengths from suffix arrays (Algorithm 1) could be made more efficient by using Burrows–Wheeler indexes instead (Mäkinen *et al.* 2023, ch. 9). Burrows–Wheeler indexes have recently taken center stage in string algorithmics yielding substantial gains in time and especially space compared to classical approaches based on suffix arrays (Cunial *et al.* 2022, Rossi *et al.* 2022). We used enhanced suffix arrays in this work, as this simplified the implementation of Algorithm 1. However, we plan to explore Burrows–Wheeler indexes in future versions of Fur.

Apart from being efficient, a good marker tool also needs to be accurate. Both Fur and KEC were highly accurate on simulated 5-Mb sequences (Fig. 4). High accuracy on simulated data is necessary for accurate analysis of real data. The only caveat with Fur is that as a rule of thumb, the length of the sliding window is the length of the shortest marker that can be detected (Fig. 4). This might suggest that we should choose very short windows, perhaps of length 1, to be on the safe side. However, very short windows do not contain enough information for assessing the average match length, the basis for deciding whether or not a window is unique. We found the default window length of 80 bp to be a good compromise.

Establishing the efficiency and accuracy of Fur opened the way to applying it to large samples of genomes. To make this feasible, we wrote supporting software for finding genomes (Neighbors) and for evaluating markers (Prim). We designed Neighbors and Prim as independent of Fur. In this way, users of Fur can generate its input and evaluate its output according to their preferences.

Picking the input automatically turned out to be much harder than checking the output. The reason for this is that genomes come with taxonomic information, but the separation between targets and neighbors needs to be phylogenetically correct for modern marker discovery to work (SantaLucia *et al.* 2020). As a result, we first pick target and neighbor genomes from the taxonomy and then sort them into the final target and neighbor samples based on their whole-genome phylogeny.

With Neighbors, Fur and Prim in place, we analyzed 120 reference prokaryotes and then concentrated on the 10 most sequenced taxa among them (Table 1). Some of the samples in the top 10 were rather large. For example, the 1460 neighbor genomes of *Streptococcus epidermidis* amounted to 4.1 Gb of sequence data. Calculating its database with the older version of Fur on our server would have required 260.7 GB of memory and 1 h, 35 m, 15 s of time. In contrast, the new Fur just uses 4.1 GB of RAM and 4 m 35 s run time on our testing laptop in default multi-threaded mode.

We restricted our data analysis to bacteria as this was the use case that had originally motivated the development of Fur. However, Fur could also be used for marker discovery in viruses and, given enough memory, eukaryotes, including mammals. In fact, we are currently using Fur to investigate lineage-specific indels in mice.

The markers output by Fur often contained a large number of Ns (Table 2). This is because even a singleton mutation among the targets currently leads to an N in the marker sequence. The expected number of mutations in a sample of sequences grows with the logarithm of the sample size, i.e. beyond all bounds (Watterson 1975). So, we are currently working on a version of Fur that allows a minimum allele frequency to be set for the minor allele of a mutation. This could be useful if the Ns are too dense for finding primers.

In six of the 10 taxa investigated in detail, we found markers in genes encoding hypothetical proteins (Supplementary Table S1). This illustrates the limitations of any candidate gene approach. As mentioned above, Limberis and Metcalfe (2023) designed multiplexed diagnostic primers for *M.tuberculosis* using eight antibiotic resistance genes. However, our single marker amplicon is located in a hypothetical gene, i.e. in a gene that could not have been picked as a marker candidate. The extra effort required to analyze whole genomes is clearly justified.

The excellent *in silico* sensitivity and specificity of the primers obtained from our markers (Table 3) is contingent on the current contents of a sequence database like nt and may change in the future. However, a low specificity like the 0.72 found for *S.enterica* should, of course, lead to the rejection of the primers. In this particular case, we could discard the marker that intersects the phage and repeat the primer design.

To sum up, we have shown that the new Fur is an efficient and accurate tool for picking markers. In conjunction with Neighbors to find its input and Prim to evaluate its output, Fur is ready to be used for marker discovery in the large.

## Supplementary Material

vbae113_Supplementary_Data

## Data Availability

The data underlying this article are available as a Code Ocean capsule at https://doi.org/10.24433/CO.7955947.v1.
