# Supplementary material for: Marker discovery in the large
Source: Bioinform Adv. 2024 Jul 27;4(1):vbae113. doi: 10.1093/bioadv/vbae113 (PMC11310107; doi:10.1093/bioadv/vbae113)
Supplement: vbae113_Supplementary_Data [file vbae113_supplementary_data.pdf]

Supplementary Material for *Marker Discovery in the Large*

Beatriz Vieira Mourato[1], Ivan Tsers[1], Svenja Denker[1,2], Fabian Klötzl[3], and Bernhard Haubold[1]

[1]Research Group Bioinformatics, Max-Planck-Institute for Evolutionary Biology, Plön, Germany
[2]Lübeck University, Germany
[3]Illumina Ltd., Cambridge, UK

June 20, 2024

Table S1: Amplicons generated by the primers in Table 3 and the annotations of the genomic regions they intersect.

#	Species	Accession	Amplicon	Annotation
1	*P. aeruginosa*	AE004091	3,137,843–3,137,966	hypothetical protein
2	*B. pertussis*	BX470248	3,597,174–3,597,290	putative degenerate prophage
3	*M. tuberculosis*	AL123456	2,981,827–2,981,974	hypothetical protein
4	*E. faecium*	CP003583	506,285–506,403	hypothetical protein
5	*P. plantarum*	AL935263	2,669,678–2,669,802	cell surface protein precursor LPXTG-motif cell wall anchor
6	*E. faecalis*	AE016830	227,001–227,087	maltose O-acetyltransferase, putative transcriptional regulator
7	*S. pneumoniae*	AE007317	1,810,602–1,810,725	phosphotransferase system system, cellobiose-specific IIA component
8	*S. epidermidis*	AE015929	461,299–461,194	conserved hypothetical protein
9	*S. enterica*	AL513382	2,760,516–2,760,604	hypothetical protein, lysogenic bacteriophage
10	*B. pseudomallei*	BX571965	1,618,130–1,618,255	hypothetical protein
